# Impact of 10 Weeks of Yoga Intervention on Mental Health and Overall Well-Being Among Medical Students: GSY Study

**DOI:** 10.3390/sports13040114

**Published:** 2025-04-10

**Authors:** Shalini Chauhan, Sachal Sadiq Najaf, Gergely Lukács, Király Anita Kinga, István Karsai, Viktoria Prémusz

**Affiliations:** 1 Doctoral School of Health Sciences, Faculty of Health Sciences, University of Pécs, H-7621 Pécs, Hungary; lukacsg12@gmail.com (G.L.); kiralyanita.phd@gmail.com (K.A.K.); istvan.karsai@aok.pte.hu (I.K.); premusz.viktoria@pte.hu (V.P.); 2 Physical Education and Exercise Center, Medical School, University of Pécs, H-7624 Pécs, Hungary; 3 GSY—Goodbye Stress with Yoga Project, University of Pécs, H-7621 Pécs, Hungary; najaf.sachal@edu.pte.hu; 4 Evidence-Based Medicine, Epistudia, 3008 Bern, Switzerland; 5 Institute of Psychology, Faculty of Humanities and Social Sciences, University of Pécs, H-7624 Pécs, Hungary; 6 National Laboratory on Human Reproduction, University of Pécs, H-7624 Pécs, Hungary; 7 Institute of Physiotherapy and Sports Science, Faculty of Health Sciences, University of Pécs, H-7621 Pécs, Hungary; 8 Physical Activity Research Group, János Szentágothai Research Center, University of Pécs, H-7624 Pécs, Hungary

**Keywords:** yoga, medical students, mental health, well-being, quality of life, quality of sleep

## Abstract

Background: the purpose of this study was to explore the effect of 10 weeks of yoga intervention on the mental health outcomes (stress, anxiety, and depression), quality of life, emotional regulation, and quality of sleep of medical students. Method: In the current experimental study, 220 medical students, with a mean age of 21.36 ± 2.20 years, participated in a 10-week yoga intervention at the University of Pécs. Data were collected before and after the intervention using the validated questionnaires DASS-21, WHOQOL-BREF, PSQI, and DERS. The distribution of data was checked using the Kolmogorov–Smirnov test. A paired sample T-test was used to compare the mean of the continuous variable. Stepwise linear regression was used to assess the association between mental health outcomes and quality-of-life variables. Results: The present study shows a significant reduction in (*p* < 0.001), depression (*p* < 0.001), and anxiety (*p* < 0.001) for participants, and overall quality of life (*p* < 0.001), quality of sleep (*p* < 0.001), and emotional regulation (*p* < 0.001) significantly improved after the intervention. The stepwise linear regression shows a strong association between higher levels of anxiety (β = 0.608, R^2^ = 0.366) and depression (β = 0.608, R^2^ = 0.392), with higher stress levels and improvement in environmental conditions being associated with a decrease in stress levels (β = −0.392, R^2^ = 0.087). Conclusions: the current study shows that yoga significantly improved the mental health and well-being of medical students, improving quality of life, quality of sleep, and emotional regulation. Registered Clinical Trial: NCT06661603.

## 1. Introduction

In the last decade, more attention has been given to the mental health issues faced by medical students [1]. It has been proven by many studies that at the beginning of their education, medical students face the same level of stress as non-medical students [2,3]. Still, evidence shows that medical student’s mental health deteriorates throughout medical education and training [4,5,6]. Medical students face a continuously demanding environment that often leads to poor sleep, high levels of stress, low levels of quality of life, and emotional dysregulation [7,8]. A recent systematic review by Shafiee et al. (2024) states that approximately half of medical students suffer from some type of sleep disturbance, anxiety, and depression problems [9].

The amount of stress on medical students is an especially concerning issue as it not only disturbs their professional development and academic performance but also affects their mental and physical health, which can lead to long-term chronic disease [10,11,12,13]. Chronic stress and symptoms of anxiety can disturb cognitive function, lead to burnout, and hinder decision making, which collectively affects the quality of care these students will provide to patients [14]. Evidence shows that the top four reasons for high levels of stress were time pressure, excessive study material, examinations, and the thought of falling behind in work [15]. However, it has been proven that medical students and doctors are at very high risk of mental health problems and chronic health issues, but increased attention has been given to the well-being and mental health of doctors and health professionals, while not much attention has been given to medical students [15].

Studies show that educational institutes face challenges in providing stress management techniques [16]. These data are concerning, as the first year of study is the foundational year of medical student’s education and is crucial for building up the coping strategies and resilience, they will require during their professional lives [17]. Additionally, these students suffer more mental health challenges as they are confronted with topics like grief and death, which leads to depression in medical students [2]. Data from German studies demonstrate that quality of life, which is related to health, is reduced, and higher levels of depression are found in medical students [18]. A study conducted by Saravanan and Wilks [19] shows that there is a direct and significant relationship between depression and stress. The prevalence of sedentary behavior among students is higher [20]. Medical students show higher levels of sedentary behavior, leading to sleepiness [21]. It has been proven that a higher level of sedentary behavior is a risk factor for chronic diseases such as diabetes, cardiovascular disease, etc. [22]. This evidence shows that high-stress environments negatively influence emotional discomfort and the overall well-being of medical students [23,24]. Yoga and meditation interventions are known to improve mental health by reducing the symptoms of depression and anxiety. Research shows that yoga and meditation interventions positively impact individuals’ overall well-being [25,26,27,28]. Yoga comes from the Sanskrit word “yuj”, which means to join or to unite. Regular yoga and meditation practice promotes endurance, flexibility, and strength and cultivates calmness and well-being [16,28]. Yoga is known as mind–body medicine, and it involves an individual’s mental, physical, and spiritual components to improve overall health and well-being [29]. It has been proven that 10 weeks of yoga and meditation practice can decrease stress-related illness such as heart disease, stroke, cancer, and diabetes, as well as many other chronic diseases [30].

However, the existing studies explore the prevalence of chronic stress and its expected consequences, but limited evidence is available where it has examined the structured interventions that target reduction in stress and overall improvement in the health and well-being of medical students.

Understanding that medical students are continuously under chronic stress, the present study employs a rigorous methodological approach to decrease the level of publication bias. This study’s primary aim is to explore the effect of a 10-week yoga intervention on the mental health outcomes (stress, anxiety, and depression), quality of life, emotional regulation, and quality of sleep of medical students. The findings should assist medical students and educational institutes in enhancing stress management techniques and influence overall well-being.

## 2. Materials and Methods

This study was reviewed and approved by the Regional Research Ethics Committee under the Institutional Review Board record number 9117-PTE 2022 of the University of Pecs. In the present study, participants were medical faculty students currently enrolled at the University of Pécs, Hungary, who were selected based on non-random convenient sampling, as the students registered for the yoga course voluntarily. Due to the nature of the selection criteria, an active control group was not included for ethical reasons. Students registered for the course voluntarily, intending to decrease their level of stress. It would not be ethically feasible to randomly assign students to the group that would not receive yoga intervention. This study was conducted between 15 February and 15 May 2023.

The current study is the first phase of the main study, which builds upon the previously conducted pilot study [16] using the same yoga protocol for 10 weeks. The duration of the intervention was selected based on evidence from numerous studies that show that physical activity training lasting between 8 weeks and 12 weeks is effective for desirable health outcomes [31,32,33,34].

### 2.1. Study Population

In total, 220 medical students with a mean age of 21.36 ± 2.20 years participated in the current study. Out of the total participants, 89.2% were female, and 10.8% were male. Students who had never practiced yoga before registered for the course, which was named “Indian Yoga”, and were encouraged to take part in the research, and the research team took their written and verbal consent. To ensure the safety of the participants, we built inclusion and exclusion criteria to select the participants to take part in the yoga intervention.

However, all participants interested in the research met the inclusion criteria. Notably, there were no dropouts (Table 1).

### 2.2. Intervention

The current study’s yoga intervention took place between 15 February and 15 May 2023. It was a 10-week (90-min-per-week) program specifically designed to reduce participants’ stress levels. The program’s structure was based on the “Goodbye Stress with Yoga Protocol” (GSY) [16], developed collaboratively by a medical researcher and a certified yoga teacher from India.

Each session of yoga intervention included the following components:

1. Stretching and warm-up—the yoga intervention session began with a complete body warm-up of stretching with breathing techniques for 10 min; this prepares an individual for further yoga exercise.
2. Asanas (yogic posture)—this includes a 50 min session of practicing yoga postures, which include standing, sitting, supine, and prone postures. (In between the sessions, participants practiced Shavasana (corpse pose), a relaxation pose that helps them absorb the session’s benefits).
3. Pranayama (breathing exercise)—this includes a 15 min breathing exercise that helps relax and improve lung capacity.
4. Meditation—the detailed meditation session is specifically designed to reduce stress levels.

Before participating, all the students were instructed not to drink or eat for two hours before the session. The students were advised not to take part in any other physical activity during the 10-week intervention. All the students confirmed in the consent form that they would not participate in any other physical activities during the intervention period. The intervention was held on the premises of the University of Pécs, in the RG room of the Faculty of Humanities. The room and mats used for intervention were cleaned and sanitized before practice to ensure the safety of the participants. The intervention was provided by a certified yoga teacher with a minimum of seven years of experience.

### 2.3. Outcome Reported

In the present study, we assessed mental health (depression, stress, and anxiety), quality of sleep, and quality of life. The tool used for mental health measurements include the depression, anxiety, and stress scores of the participants before and after the intervention. However, quality of life consists of a score of overall quality of life and a sub-score of physical health, psychological health, social relationships, and environment before and after the yoga intervention. Additionally, the secondary outcome was sedentary behavior and emotional regulation, which includes a score of nonacceptance of emotional responses, impulse control difficulties, difficulty with engaging in goal-directed behavior, lack of emotional awareness, limited access to emotional regulation strategies, and lack of emotional clarity among the participants.

### 2.4. Measures

Self-reported questionnaires were used to collect the data from the participants. All questionnaires were provided in paper-and-pencil form, and data were calculated before and after the 10-week yoga intervention. Before collecting the data, all questionnaire details were explained thoroughly to participants to reduce recall bias.

#### 2.4.1. Assessment of Mental Health

We used the English version of the Depression Anxiety Stress Scale 21 (DASS 21) [35]. This is a validated questionnaire which includes 21 questions divided into three sets to analyze the emotional states of anxiety, depression, and stress in an individual. These three sets include seven questions each for these subsections with an overall score ranging from “normal” to “extremely severe” [31].

#### 2.4.2. Assessment of Quality of Sleep

We used the English version of the Pittsburgh Sleep Quality Index (PSQI) [32]. It is a self-assessment questionnaire that records sleep disturbance and sleep quality. It has, in total, 19 questions that produce seven component scores. These seven components are sleep quality, sleep duration, sleep latency, sleep disturbances, habitual sleep efficiency, daytime dysfunction, and use of sleep medication. Combined scores from all seven components generate a global sleep quality score [32].

#### 2.4.3. Assessment of Quality of Life

We used the English version of the World Health Organization Quality of Life (WHOQOL-BREF) [33] tool. Participants reported their perception of quality of life over the last two weeks, which reduced the possibility of recall bias. The tool is self-administered, contains 26 questions, and is presented in four domains: 1. psychological, 2. physical health, 3. social relationships, and 4. environment.

#### 2.4.4. Assessment of Emotional Regulation

We used the Difficulties in Emotion Regulation Scale (DERS) [36]. This instrument has 36 self-reported questions that report how respondents relate to their emotions, specifically in six categories: nonacceptance of emotional responses, difficulty engaging in goal-directed behavior, impulse control difficulties, lack of emotional awareness, limited access to emotion regulation strategies, and lack of emotional clarity.

### 2.5. Statistical Analyses

We used SPSS 26.0 software (SPSS Inc., Chicago, IL, USA) to conduct statistical analyses. The distribution of the data was tested using the Kolmogorov–Smirnov test. Based on the distribution of the data, a paired sample T-test was conducted to compare the mean of continuous variables. The association between continuous variables was tested using Pearson’s correlation. Based on the significance of these correlations, we conducted a stepwise linear regression analysis to define the association of changes in stress level, anxiety level, and depression level with physical health and environmental factors. Statistical significance for the overall linear regression model was assessed by using the F-test and *p*-value. The collinearity diagnostics show that factors have a variance inflation factor (VIF) and tolerance value. If VIF is below 10 and tolerance values are above 0.1, this indicates no multicollinearity concerns, which shows that the selected model is well conditioned. Data were demonstrated in mean and SD for the continuous variable as well as percentage and frequency for the categorical variable. *p* ˂ 0.05 is considered significant in each case.

## 3. Results

Table 2 demonstrates the characteristics of the included participants. In total, 212 medical students participated in the current study, averaging 21.36 ± 2.20 years; 89.2% were female, and 10.8% were male.

### 3.1. Change in Reported Outcome

The mean and standard deviation of all the reported parameters can be seen in Table 3. After 10 weeks of yoga intervention, a significant decrease was seen in the mental health outcomes (depression, anxiety, and stress, *p* < 0.001), with considerable improvement in quality of life and quality of sleep (*p* < 0.001) and emotional regulation (*p* < 0.001). In the current study, 89.1% of participants were female and 10.8% were male. No significant differences were found in the reported outcomes between male and female participants, with a *p*-value ranging from *p* = 0.100 to 0.538.

### 3.2. Stepwise Linear Regression Analysis

The stepwise linear regression analysis of changes in stress, depression, and anxiety is demonstrated and summarized in Table 4, Table 5 and Table 6. An increase in depression (β = 0.626, *p* < 0.001, Adjusted R^2^ = 0.392, tolerance 0.615, VIF 1.623) and anxiety (β = 0.608, *p* < 0.001, Adjusted R^2^ = 0.366, tolerance 0.601, VIF 1.663) showed a strong positive relation to higher stress levels, while environmental condition improvement is associated with decreased stress levels (β = −0.302, *p* < 0.001, Adjusted R^2^ = 0.087, tolerance 0.941, VIF 1.063) (Table 4). Table 5 shows that a change in depression is primarily and positively associated with stress (β = 0.626, *p* < 0.001, Adjusted R^2^ = 0.392, tolerance 0.622, VIF 1.608) and anxiety (β = 0.619, *p* < 0.001, Adjusted R^2^ = 0.380, tolerance 0.577, VIF 1.734). Additionally, the small contribution to physical health (β = −0.262, *p* < 0.001, Adjusted R^2^ = 0.064, tolerance 0.909, VIF 1.101) is also associated with a decrease in depression. Table 6 shows that a change in anxiety is strongly associated with depression (β = 0.619, *p* < 0.001, Adjusted R^2^ = 0.383, tolerance 0.562, VIF 1.780) and stress (β = 0.608, *p* < 0.001, Adjusted R^2^ = 0.369, tolerance 0.561, VIF 1.782) levels. However, improvement in environmental conditions (β = −0.237, *p* < 0.001, Adjusted R^2^ = 0.052, tolerance 0.894, VIF 1.118) and physical health (β = −0.280, *p* < 0.001, Adjusted R^2^ = 0.070, tolerance 0.905, VIF 1.105) leads to a reduction in anxiety. The fitness of the model showed statistical significance for the change in stress (*p* < 0.001), change in depression (*p* < 0.001), and change in anxiety (*p* < 0.001). The collinearity diagnostics showed that all the predictors had VIF below 10 and tolerance values above 0.1; this indicates that multicollinearity is not a concern, and the model used is well conditioned.

## 4. Discussion

We explored the 10-week yoga intervention’s impact on mental health and well-being using the experimental pre–post-test intervention method. Our findings showed that a 10-week yoga intervention significantly decreases stress (*p* < 0.001), anxiety (*p* < 0.001), and depression (*p* < 0.001), which leads to improvement in the mental health of medical students. Among the participants, there were more female than male participants, and this observation is supported by the systematic review that yoga practice is greater among female participants. Another study showed that male participants reported heterosexual self-presentation as a barrier to not adopting yoga practice [37]. However, we would like to convey the message that yoga is not for a particular gender, and the findings from current study support that benefits of yoga not being dependent on a participant’s gender [38]. In the present study, the participants felt improvement in their emotional regulation, quality of sleep, and quality of life, leading to an overall improvement in well-being after 10 weeks of yoga intervention. This highlights that yoga intervention can potentially improve mental health and well-being [28]. Additionally, current findings present an interrelated relationship between mental health outcomes and psychosocial factors that shows an essential role in influencing changes in mental health outcomes.

The finding supports the idea that practicing meditation and yoga can improve quality of life and mental health, resulting in a healthy body and mind [35,36]. The results show that 10 weeks of yoga intervention significantly enhanced the medical students’ mental health. Additionally, they reveal that reducing depression and anxiety is a strong predictor of decreasing stress levels, and improvements in physical health and environmental conditions contribute to reducing anxiety levels and stress levels. This shows that yoga is a holistic intervention that not only improves mental health and well-being but also creates a calming environment that results in stress relief. The focus of yoga intervention on breathing exercises, posture with conscious breathing, and meditation relaxation techniques aligns with previous research, which demonstrates that yoga’s integrative and holistic approach leads to exploring not only physical activity but the whole journey toward reduction in stress, improvement in emotional well-being, and balanced lifestyle [37].

The finding supports the idea that practicing meditation and yoga can improve quality of life and mental health, resulting in a healthy body and mind [39,40]. The evidence supports yoga’s holistic practice and promotes better sleep quality by decreasing stress and calming the mind before bedtime [38,39,40]. In the current study, the yoga intervention was provided in the evening after 7:00 p.m., and the intervention ended with breathing and meditation techniques. Practicing in the evening can also improve the current findings, as it leads to calming the mind before bedtime. The study participants reported the benefits of the yoga intervention, as reflected by the significant PSQI score, which demonstrates better sleep duration with sleep efficacy. Controlled breathing and physical yoga poses encourage relaxation [41,42], and yoga practice helps improve parasympathetic activation and modulation of the autonomic nervous system and favors restful sleep [43].

The participants reported significant improvements in quality of life after the yoga intervention using WHOQOL scores. This tool also shows that overall quality of life, well-being, and life satisfaction significantly improved after the yoga intervention. Quality of life measured in the current study includes the sub-score of improvement in physical health, environmental condition, psychological health, and social relationships, which were all significantly improved after the yoga intervention. This is supported by the systematic review, which states that yoga is associated with a significant decrease in negative thoughts and an increase in quality of life [44]. Medical students experience intense emotions and an absence of emotional regulation that affect their performance, motivation, and overall well-being [45,46]. In the current research, medical students showed improvements in the DERS scale, which includes the sub-score of emotional awareness, regulations, emotional clarity, and impulse control; these findings highlight that yoga interventions improved participants’ ability to respond to emotion and manage emotion effectively. This is supported by findings that show that yoga has a potential effect on emotional regulation and benefits healthier mental health outcomes [47].

The strength of the current study is that it shows that the sample size provides sufficient value to investigate the impact of yoga on meaningful changes in mental health, quality of life, quality of sleep, and emotional regulation among medical students. However, several limitations should be addressed in future studies. There was no control group, so adding a current group can help decrease the expected bias and give more clarity on whether the outcome reported is due to yoga practice or another contributing factor. Future research should focus on rigorous methodological randomized control trials with long-term intervention among medical students to minimize potential bias, as seen in the current study.

## 5. Limitations of the Current Study

In the current study, a major limitation is the absence of a control group, which limits the chance to establish causality, and it can also determine if the findings are solely attributable to the 10 weeks of yoga intervention. However, the current finding shows a significant improvement in mental health, sleep quality, quality of life, and emotional regulation. Additionally, potential confounding factors were not explicitly controlled in this study, such as lifestyle factors and dietary habits that can influence the findings. Another key limitation is a lack of long-term follow-up, which prevents observation of the benefits over time. It is recommended to interpret the findings with caution. The predominance of female participants in the current study and non-random convenient sampling may limit the generalizability of the findings, so it is recommended to interpret the findings with caution. Furthermore, future studies should focus on long-term intervention with randomized control trials to improve the robustness of the findings.

## 6. Conclusions

The current study shows that a yoga intervention used in the GSY protocol can be significantly associated with medical students’ mental health and overall well-being. The findings highlight that yoga is a holistic tool to enhance quality of sleep, quality of life, emotional regulation, and mental health outcomes; however, the results of this research should be interpreted with caution by considering the points listed in Section 5. The observed improvement in the outcomes suggests that yoga intervention can be an effective practice for overall health promotion among medical students, to navigate the challenges students face, such as emotional and mental health challenges during their training. By including yoga and meditation practice in the daily lives of medical students, educational institutes can provide a more supportive environment that focuses on students’ overall health.

## Figures and Tables

**Table 1:** Criteria for selecting participants in the current study.

Criteria	Explanation
Inclusion Criteria	Medical students enrolled in the University of Pécs, Hungary, were eligible to participate in this study.
Exclusion Criteria	The following are the exclusion criteria: 1. Participants with chronic health pain conditions. 2. Participants with physical injuries. 3. Participants with severe sclerosis. 4. Participants with congenital skeletal abnormalities. 5. Participants with severe arthritis. 6. Participants with musculoskeletal abnormalities.

**Table 2:** Characteristics of study population.

Characteristics (N = 212)	Frequency (%)
Anthropometrics	
Height (cm)	167 (150–190)
Weight (kg)	64.29 ± 13.08
Mean age (years)	21.36 ± 2.20
Gender (%)	
Female	189 (89.2)
Male	23 (10.8)
Residence	
City	190 (89.6)
Capital	9 (4.2)
County side	6 (2.8)
Village	7 (3.3)
Marital	
Married	2 (0.9)
Single but living with a partner	43 (20.3)
Single and not living with a partner	167 (78.8)
Financial Education	
Family support	77 (36.3)
Scholarship	135 (63.7)
Major (%)	
1st year	66 (31.1)
2nd year	73 (34.4)
3rd year	70 (33.0)
4th year	3 (1.4)

Note: cm—centimeter; kg—kilogram; kg/m^2^—kg/square meter.

**Table 3:** Mean and standard deviation of participants’ mental health and well-being after 10 weeks of yoga intervention.

N = 212	Pre	Pre	Post	Post	Change	Change	Significance
Variables	Mean	SD	Mean	SD	Mean Diff.	SD	Two-Sided *p*
Mental Health							
Depression	13.04	8.90	5.26	4.39	−8.00	6.96	<0.001
Anxiety	14.13	8.22	5.22	4.33	−8.90	6.40	<0.001
Stress	14.20	8.06	5.67	4.39	−8.52	6.35	<0.001
Quality of Life							
Overall Quality of Life	5.59	1.30	8.33	0.89	2.74	1.53	<0.001
Physical Health	23.06	2.42	24.54	1.94	1.47	3.03	<0.001
Psychological Health	16.19	1.89	20.88	2.07	4.69	2.85	<0.001
Social Relation	9.15	1.72	12.33	1.48	3.18	1.94	<0.001
Environment	29.13	3.52	32.06	3.40	2.92	4.18	<0.001
Quality of Sleep (PSQI)							
Global Quality of Sleep Score (PSQI)	128.23	24.19	115.04	15.16	−13.19	28.29	<0.001
Emotional Regulation (DERS)							
Nonacceptance of emotional responses	18.16	3.84	11.79	3.69	−6.36	5.02	<0.001
Difficulty engaging in goal-directed behavior	13.47	2.90	10.89	2.90	−2.58	3.46	<0.001
Impulse control difficulties	16.77	3.50	11.82	3.10	−4.95	4.09	<0.001
Lack of emotional awareness	16.17	3.73	17.33	4.39	1.16	5.11	0.001
Limited access to emotion regulation strategies	19.53	4.26	16.62	3.54	−2.90	4.65	<0.001
Lack of emotional clarity	14.99	3.14	10.49	1.93	−4.50	3.31	0.001

Note: Significant at *p* < 0.005; SD, standard deviation.

**Table 4:** Stepwise regression investigating which factors contribute most to changes in stress.

Empty Cell	Β	SE	*β*	*p*-Value	Adjusted R^2^
Change in Anxiety	0.602	0.054	0.608	<0.001	0.366
Change in Depression	0.571	0.049	0.626	<0.001	0.392
Change in Environmental Condition	−0.459	0.100	−0.302	<0.001	0.087

Note: Β = unstandardized coefficient; SE = standard error; *β* = standardized coefficient; *p*-value indicates statistical significance; Adjusted R^2^ reflects explained variance in the outcome variable by each predictor. *p* < 0.001 is significant.

**Table 5:** Stepwise regression investigating which factors contribute most to changes in depression.

Empty Cell	Β	SE	*β*	*p*-Value	Adjusted R^2^
Change in Stress	0.687	0.059	0.626	<0.001	0.392
Change in Anxiety	0.673	0.059	0.619	<0.001	0.380
Change in Physical Health	−0.601	0.153	−0.262	<0.001	0.064

Note: Β = unstandardized coefficient; SE = standard error; *β* = standardized coefficient; *p*-value indicates statistical significance; Adjusted R^2^ reflects explained variance in the outcome variable by each predictor. *p* < 0.001 is significant.

**Table 6:** Stepwise regression investigating which factors contribute most to changes in anxiety.

Empty Cell	Β	SE	*β*	*p*-Value	Adjusted R^2^
Change Depression	−4.349	0.528	0.619	<0.001	0.383
Change Stress	0.613	0.055	0.608	<0.001	0.369
Change in Physical Health	−0.591	0.140	−0.280	<0.001	0.070
Change in Environmental Condition	−0.363	0.103	−0.237	<0.001	0.052

Note: Β = unstandardized coefficient; SE = standard error; *β* = standardized coefficient; *p*-value indicates statistical significance; Adjusted R^2^ reflects explained variance in the outcome variable by each predictor. *p* < 0.001 is significant.

## Data Availability

Data are available upon reasonable request from the authors.
